# Platelet-rich plasma versus corticosteroid: a randomized controlled trial on tennis elbow patients resistant to nonsurgical treatments

**DOI:** 10.1097/MS9.0000000000001115

**Published:** 2023-08-03

**Authors:** Shahram Sayadi, Parmida Shahbazi, Arvin Najafi, Fatemeh Ochi, Kyana Jafarabady, Mohammad M. Rezaei, Salman Azarsina

**Affiliations:** a Anesthesiology Research Center, Shahid Beheshti University of Medical Sciences; b Orthopedic Department, Orthopedic Surgery Research Center (OSRC), Sina Hospital, Tehran University of Medical Sciences, Tehran; c Department of Orthopedic Surgery, Shahid Madani Hospital, Alborz University of Medical Sciences; d Student Research Committee, Alborz University of Medical Sciences, Karaj, Iran

**Keywords:** corticosteroids, platelet-rich plasma, randomized controlled trials, tennis elbow

## Abstract

**Background:**

Although some studies on tennis elbow indicate corticosteroid (CS) effectiveness in the short term, according to the role of race, this study evaluates the efficacy of platelet-rich plasma (PRP) compared with CS for a more cost-effective treatment.

**Methods:**

This randomized controlled trial included 30 positive-resisted wrist extension patients with a minimum five visual analog scale (VAS) pain score. Participants were randomly assigned to treatment or control groups via computer-generated randomization and were matched for baseline and clinical characteristics. Cases received either 40 mg of prednisolone acetate or 2 ml of PRP, followed for 1 month. VAS and Disabilities of the Arm, Shoulder, and Hand (DASH) scores were the primary outcomes.

**Results:**

The median VAS and the mean DASH scores had a statistically significant difference in the PRP and CS groups before and after injection (*P*<0.001).

The mean DASH difference between preinjection and follow-up time in the PRP and CS groups was 59.72±14.17 and 43.16±10.87, respectively, with a mean difference of 16.55 (95% CI 7.10–26.00) and a significant difference (*P*=0.001).

The mean VAS pain score difference in preinjection and follow-up time had a statistically significant difference between the PRP and CS groups (*P*=0.026), and the mean VAS pain score difference in the CS group was 6.46±1.50 and 7.73±0.96 in the PRP group.

**Conclusion:**

In conclusion, larger studies with parallel groups and more diverse CS doses and types with baseline matching are needed to confirm the short-term benefits of PRP. Investigating the effects of different CS doses using ultrasound techniques is recommended.

## Introduction

Highlights

- Tennis elbow is a common tendinosis inflammatory disorder.
- The short-term efficacy of platelet-rich plasma compared with corticosteroid (CS) for more cost-effective treatment is challenging yet.
- The recurrence of symptoms and reduced tendon thickness in the use of CS have been reported. However, platelet-rich plasma is significantly more expensive.
- Studies with more parallel groups in a higher sample size in different doses and types of CS with baseline matching and investigating the CS side effects and tendon degeneration in different doses with ultrasound techniques are recommended.

Lateral epicondylitis (LE), or tennis elbow, is a common tendinosis inflammatory disorder with a prevalence rate of 1–3% that affects the population^1^. In a previous population-based study, the incidence rate was reported between 40 and 49 years old, with a lower incidence in men^2^ and a higher severity with a longer duration of symptoms in women^3^.

Despite surgical treatments as the last option, due to their evidence insufficiency^1^ widely, nonsurgical treatments are applied to reduce pain and inflammation. Such as resting, bracing, physical therapy, nonsteroidal anti-inflammatory drugs (NSAIDs), corticosteroid (CS) injections, extracorporeal shock wave therapy, botulinum toxin injection, and whole blood and platelet-rich plasma (PRP)^4–6^.

Few studies have addressed the role of race and genetic factors as risk factors for LE and elbow tendon pathologies^7,8^. Moreover, the cost of PRP injections compared to CS injections in Iran is five times higher and is not covered by insurance plans.

With these in mind, this study evaluates the efficacy of the PRP compared with CS 1 month after injection for the first time in Iran to use a more cost-effective treatment for the patients.

## Methods

This randomized two-arm parallel-group controlled trial was conducted on patients referred to the hospital orthopedic clinic who were resistant to the LE nonsurgical treatments from May 2020 to November 2020 and had informed consent.

All patients were diagnosed based on a positive resisted wrist extension test (extension of the affected arm by making a fist, then rotating forearm inward and bending the wrist toward it) and a minimum five visual analog scale (VAS) pain score.

The eligibility criteria included the patients who not only did not receive any surgical treatments or CS but also had chronic (≥6 weeks and ≤1 year) symptoms despite using nonsurgical conservative treatments, including rest, avoiding strenuous activities, physical therapy, NSAIDs, and life-changing.

The exclusion criteria were patients older than 70 years old, patients having a systemic disease (ischemic heart disease, diabetes, rheumatoid arthritis, and hepatitis), positive autoimmune disease history, infectious arthropathies, malignancy, radiculopathy, peripheral nerve deficit, use of antiplatelets 10 days before injection or NSAIDs 48 h before injection, steroid applied within 3 last weeks, positive history of vasovagal shock, and women ascertained or suspected pregnancy or lactating.

Forty milligrams of prednisolone acetate with a volume of 1 ml were drawn into a 22-gage needle syringe and injected into the control group at the maximum tenderness point of the elbow by peppering injecting technique. Two milliliter of PRP obtained from the patient’s blood centrifuge were injected into the intervention group using a peppering technique with a 22-gage needle syringe at the maximum tenderness point. Unfortunately, blinding could not be done due to blood sampling in just one of the groups (the intervention group). Eight minutes before the injection, the prepped and draped injection site skin was anesthetized by injecting 2 ml of 5% lidocaine. No CSs or nonsteroidal anti-inflammatory drugs were prescribed during the follow-up period.

### PRP preparation

In the PRP group, 20 ml of blood was taken from the patient and placed in a special collection kit. Using one’s blood limits the risk of transmission of blood-borne diseases. This kit was centrifuged for 15 min at a speed of 1600 revolutions per minute (rpm) to separate the erythrocytes and then for 7 min at 2800 rpm to concentrate the platelets.

The pain was assessed based on the VAS pain score. The function was evaluated by a Disabilities of the Arm, Shoulder, and Hand (DASH) score, a 30-item self-reported questionnaire on two levels, before the interventions and a month after that in clinical visits. The other gathered data included age, sex, and patients’ job from history taking.

The sample size was convenient and calculated based on previous studies.

### Randomization

Participants were randomly assigned to either the treatment or control group using a computer-generated randomization process. All participants were matched for baseline and clinical characteristics, including age, sex, occupation, duration of the symptoms, preinjection VAS, and preinjection DASH.

### Statistical analysis

Categorical variables were described as frequencies, and continuous variables were described using the mean standard deviation (SD), median, and range. Proportions for categorical variables were compared using the *χ*^2^-test. An independent samples *t*-test or a Mann–Whitney *U* test was used to compare the numeric variable. Also, for each group’s difference between preinjection and postinjection, a paired samples *t*-test or a Wilcoxon signed-rank test was used. The SPSS application (Version 16) for Windows (SPSS Inc.) was used to analyze the data. A *P*-value less than 0.05 was considered statistically significant.

### Ethical considerations

A random computer-generated unique code identified all patients, and no personal data was revealed to any party during data extraction or analysis. All participants in this study had informed consent. This study was approved by the ethics committee of Alborz University of Medical Sciences under the IR.ABZUMS.REC.1399.036 code and the Iranian Registry of Clinical Trials (IRCT) with the IRCT registration number: IRCT20191105045335N1.

Overall, our study followed the CONSORT guidelines^9^ and adhered to ethical considerations, ensuring that our findings are transparent, reliable, and comparable to other studies in the field.

## Results

**Figure 1:**
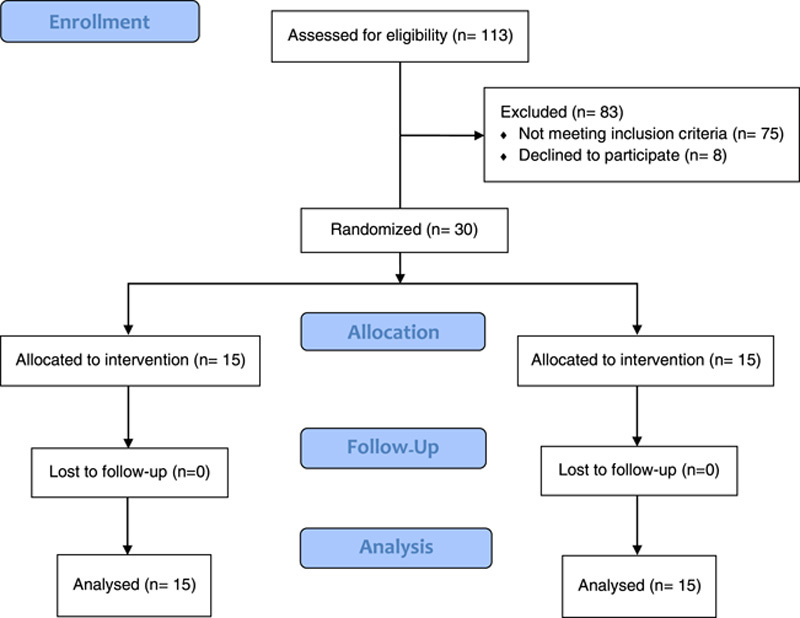
CONSORT flowchart of the present Randomized Controlled Trial.

All 30 eligible patients were included in this study from May 2020 to November 2020. They were randomly allocated into two 15-patient groups and were matched for baseline and clinical characteristics. One-month follow-up after recruitment of each case was done, and there was no loss to follow-up (Fig. 1).

The mean age was 48.97±11.64 years and 76.6% (*n*=23) of the participants were female. Of all cases, 46.7% (*n*=14) were housekeepers, followed by 30% (*n*=9) and 6.7% (*n*=2) who were an employee and retired, respectively.

The mean age in the PRP group was 47.67±11 years and 50.27±12 years in the CS group, and there was no significant difference between them (*P*=0.55). In the PRP group, 80% of the cases were female, and 73% for the CS group without any significant difference (*P*=1.00).

The median VAS score in the PRP group was 9±range (10–2) and 1±range (4–1) for preinjection and 1 month later, respectively. This proportion in the CS arm was 9±range (9–8) and 1±range (4–1) in preinjection time and follow-up, respectively. There was a statistically significant difference in VAS pain scores in both PRP and CS groups before and after injection (*P*<0.001) (Table 1).

**Table 1:** Baseline and clinical characteristics for platelet-rich plasma versus corticosteroids, preinjection, and after 1 month of follow-up.

Characteristics	PRP^1^ (*n*=15)			CS^2^ (*n*=15)			*P* ^3^
Age (years), mean±SD		47.67±11			50.27±12		0.55
Sex (female)		12 (80%)			11 (73%)		1.00
	Preinjection	After one M^8^	*P*-value^9^	Preinjection	After one M^8^	*P*-value^10^	
VAS^4^, median±range	9±(10–2)	1±(4–1)	<0.001	9±(9–8)	1±(4–1)	<0.001	
DASH^5^, mean score±SD	78.55±8.82	18.83±8.92	<0.001	74.17±6.06	31.00±7.81	<0.001	
Δ VAS^6^, mean score±SD		7.73±0.96		6.46±1.50			0.026
Δ DASH^7^, mean score±SD	59.72±14.17			43.16±10.87			0.001

1 Platelet-rich plasma.

2 Corticosteroids.

3 The comparisons were made between the patients in the PRP group and the CS group.

4 Visual analog scale pain score.

5 Disabilities of the Arm, Shoulder, and Hand score.

6 Mean VAS difference between preinjection and a month later.

7 Mean DASH difference between preinjection and a month later.

8 Month.

9 The comparison was made in the PRP group.

10 The comparison was made in the CS group.

The mean DASH score has a statistically significant difference in the PRP group (*P*<0.001) with a mean of 78.55±8.82 and 18.83±8.92 for preinjection and a month after injection, respectively. As well as the mean DASH score in the preinjection CS group was 74.17±6.06 and 31.00±7.81 in the follow-up, with a statistically significant difference (*P*<0.001).

The mean DASH difference between preinjection and follow-up time in the PRP group was 59.72±14.17 but in the CS group was 43.16±10.87 with a mean difference of 16.55 (95% CI 7.10–26.00). There was a significant difference between the PRP and CS groups (*P*=0.001).

The mean VAS pain score difference in preinjection and follow-up time had a statistically significant difference between the PRP and CS groups (*P*=0.026), and the mean VAS pain score difference in the CS group was 6.46±1.50 and in the PRP group was 7.73±0.96.

## Discussion

This study found that PRP injections were more effective than CS injections in reducing VAS pain and DASH scores after 1 month, although both treatments resulted in improved outcomes (either in pain or function) compared to preinjection scores.

Previous research found that the CS group had less pain in the short term^10^, which may have been due to a lower baseline DASH score in that group. However, our study did not find a significant difference in baseline DASH scores between the two groups, as they were matched from the beginning.

While some studies have shown that CS is more effective than PRP in the short term, others have found that PRP has greater long-term improvements in function and pain^11–14^. However, some studies have reported no significant difference between the two treatments^10,15^, which may be due to variations in the dosage or type of PRP or CS used in different studies.

Consistent with previous studies, the majority of participants in our study were female (76.6%), but we cannot consider gender as a risk factor^2,7^.

Although our study had limitations in sample size and the inability to investigate CS side effects with tendon ultrasonography, we had excellent control with baseline matching and minimized recall bias through short follow-up and data collection by a trained physician.

In conclusion, while PRP had better short-term outcomes, it is significantly more expensive than CS. Previous studies have suggested that CS may be more effective in the short term, but there are reports of symptom recurrence and reduced tendon thickness with CS use^4,16,17^.

Our study highlights the need for further research with more parallel groups in larger sample sizes and different doses/types of CS with baseline matching, as well as investigating CS side effects and tendon degeneration using ultrasound techniques.

## Ethical approval

Ethical approval for this study was provided by the Alborz University of Medical Sciences under IR.ABZUMS.REC.1399.036 code on 05 March 2020.

## Consent

Written informed consent was obtained from the patients for publication and any accompanying images. A copy of the written consent is available for review by the Editor-in-Chief of this journal on request.

## Sources of funding

Not applicable.

## Author contribution

S.S.: conceptualization, writing – original draft, writing – review and editing, and final approval; P.S.: methodology, formal analysis, writing – original draft, writing – review and editing, and final approval; A.N.: supervision, interpretation of data, writing – original draft, writing – review and editing, and final approval; F.O., K.J., and M.M.R.: investigation, formal analysis, writing – original draft, writing – review and editing, and final approval; S.A.: conceptualization, interpretation of data, writing – original draft, writing – review and editing, and final approval.

## Conflicts of interest disclosure

The authors declare that they have no conflicts of interest.

## Research registration unique identifying number (UIN)

1. Name of the registry: Iranian Registry of Clinical Trials (IRCT).
2. Unique identifying number or registration ID: IRCT20191105045335N1.
3. Hyperlink to your specific registration (must be publicly accessible and will be checked): https://en.irct.ir/trial/43577.

## Guarantor

Salman Azarsina.

## Data availability statement

Available upon reasonable request.

## Provenance and peer review

Not commissioned, externally peer-reviewed.
